# Treating Spontaneous Intracranial Hypotension with an Anesthetic Modality: The Role of the Epidural Blood Patch †

**DOI:** 10.3390/life12081109

**Published:** 2022-07-23

**Authors:** Zoi Masourou, Nikolaos Papagiannakis, Georgios Mantzikopoulos, Dimos-Dimitrios Mitsikostas, Kassiani Theodoraki

**Affiliations:** 1 Department of Anesthesiology, Aretaieion University Hospital, National and Kapodistrian University of Athens, 11528 Athens, Greece; zwhmas@hotmail.com; 2 Department of Neurology, Aiginiteion University Hospital, National and Kapodistrian University of Athens, 11528 Athens, Greece; nikolas.papagia@gmail.com (N.P.); dimosmitsikostas@gmail.com (D.-D.M.); 3 Department of Radiology, KAT General Hospital of Athens, 14561 Kifissia, Greece; gkmantzik@yahoo.gr

**Keywords:** brain, intracranial hypotension, epidural blood patch, magnetic resonance imaging, cerebrospinal fluid leak

## Abstract

**Background**: Spontaneous intracranial hypotension (SIH) is a rare syndrome characterized by heterogeneity of presentation and prognosis, which can occasionally result in serious complications, such as the formation of subdural hematomas (SDHs). This case series aims to emphasize that SIH remains a diagnostic and therapeutic challenge; it can present with a broad clinical spectrum of symptoms, can lead to SDH and, if conservative treatment fails, an epidural blood patch (EBP) is a viable treatment option. Although the exact etiology of SIH is not known, it is believed to be due to cerebrospinal fluid (CSF) leak or a low CSF pressure. **Case Series**: Three patients (two males and one female) with ages ranging between 38 and 53 years old who presented with complaints of not only an orthostatic headache, but also a variety of symptoms of SIH, including the formation of two SDHs in one of them, were included in this series. These patients did not respond to conservative management and, subsequently, given the clinical and radiological evidence of SIH, were referred to the Anesthesiology Department for an EBP. Diagnostic workup was facilitated by imaging modalities, including magnetic resonance imaging (MRI) of the brain and spinal cord, prior to the EBP. All three patients were subjected to an EBP with an 18-gauge epidural needle. A total of between 30 and 43 mL of autologous blood was collected from the patients and was injected into the epidural space under strict aseptic conditions. Two lumbar (L_1_–L_2_, L_2_–L_3_) EBPs and one thoracic (T_11_–T_12_) EBP were performed on the three patients, respectively. All patients reported complete resolution of symptoms following the EBPs, while MRI improved substantially. **Conclusions**: This report describes three cases of SIH with CSF leak originating from the cervical, the thoracic and the lumbar level. The EBP restored CSF pressure and relieved the patients’ persistent symptoms. MRI helps in revealing indirect signs of a low volume of CSF, though it may not be possible to locate the actual site of the leak. In conclusion, EBP is a well-accepted and beneficial treatment modality for SIH when conventional measures fail.

## 1. Introduction

Spontaneous intracranial hypotension (SIH) is a rare syndrome; however, it is highly disabling and characterized by heterogeneity of presentation and prognosis. The hallmark of symptoms is an acute orthostatic headache, but a broad spectrum of clinical manifestations, such as neck pain or stiffness, nausea, tinnitus or photophobia, can also be present. Despite its infrequent occurrence, the increased accessibility to and development of neurological imaging examination, such as a spine magnetic resonance imaging (MRI) and computed tomographic myelography, identify more and more recognized cases [1]. Although the exact etiology of SIH remains undetermined and has led to a series of misconceptions, it is believed to be related to cerebrospinal fluid (CSF) leak or a low CSF pressure [2]. Occasionally, in patients with well recognized SIH symptoms and typical cranial imaging findings, there is no evidence of CSF leak despite a thorough and targeted diagnostic workup. In these cases, a pathological cranial-to-spinal shift without overt CSF leak has been advocated [3]. This may be due to increased spinal compliance, leading to downward displacement of cranial structures and to the clinical manifestations of SIH. If conservative therapy is ineffective to relieve the patient’s symptoms, then the mainstay of therapy is an epidural blood patch (EBP), wherein the anesthesiologist is involved [4]. On some occasions, CSF leaks may be amenable to surgical treatment, as in the case of dural tears, ruptured meningeal diverticula or CSF-to-venous fistulae [5,6,7]. The diagnosis of SIH is often missed due to a lack of awareness or consideration of the diagnosis. We present a case series of three patients with a variety of clinical features of SIH; they had complaints of not only a headache, which is the classic and most common symptom of the syndrome, but also of dizziness they could not tolerate. A provisional diagnosis of SIH was made and was confirmed by the findings on MRI. Thereafter, the patients were treated successfully with an EBP and remained asymptomatic after the procedure on the follow-up. This case series aims to emphasize that SIH remains a cryptic diagnosis; it can present with a broad clinical spectrum of symptoms, diagnosis is mainly based on the severity of the clinical features of the patient and the magnetic resonance of the brain and spine complete the diagnostic procedure. It can cause subdural hematoma (SDH) and, if conservative treatment fails, the mainstay of therapy remains an EBP. 

## 2. Cases Description

### 2.1. Patient I 

A 53-year-old male presented to the Neurology Outpatient Department with complaints of episodes of dizziness over the last month, with no signs of vertigo but accompanied by hearing disturbance. He could also sense the onset of dizziness at an earlier stage during each episode along with a feeling of instability accompanied by headache, which was worse while sitting up, but was relieved on lying down. There was no loss of consciousness, no history of craniospinal trauma or recent central neuraxial blockade. On clinical examination, no neurological deficits were elicited and no papilledema was detected. On further neurological examination, the level of consciousness, papillary light reflex and the muscle force of limbs were intact, but mild dysarthria and muscular weakness of the left lower extremity were revealed.

**Figure 1:**
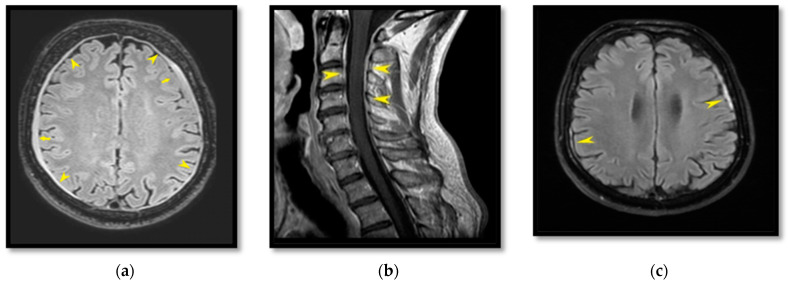
Flair sequence in transverse plane; diffuse pachymeningeal reinforcement (yellow arrow heads) and two emerging subdural hematomas: one left frontal and one right temporal–parietal (small yellow arrows) (**a**). T_1_W sequence in sagittal plane after intravenous contrast medium (gadolinium–diethylenetriaminepentaacetic acid complex (Gd–DTPA)) at the cervical level; homogeneous meningeal reinforcement (yellow arrow heads) is highlighted (**b**). Flair sequence in transverse plane one month after the initial examination; clear decrease in the thickness of the subdural hematomas on the left frontal and the right temporal–parietal region, compared with the previous examination before the EBP (**c**).

MRI study (T_1_-weighted (T_1_W) sequence in the transverse plane after intravenous contrast medium and a flair sequence in the transverse plane) showed an excess of pachymeningeal enhancement while two subdural hematomas were detected on the flair sequence: one left frontal (5.5 cm × 6 mm) and a second right temporal–parietal (5.3 cm × 3 mm) (Figure 1a). Spinal imaging (T_2_-weighted (T_2_W) sequence in the sagittal plane) also showed evidence of two CSF leaks by revealing one fluid collection at the anterior cervical level and another one at the L_2_–L_3_ lumbar level. The T_1_W sequence in the sagittal plane after intravenous contrast medium also demonstrated homogenous pachymeningeal enhancement at the cervical spinal level (Figure 1b). Imaging features were consistent with the diagnosis of SIH.

The electroencephalogram was normal. A lumbar puncture test showed that the CSF pressure was 6 cmH_2_O. Given the clinical and radiological evidence of SIH, the patient was referred to the Anesthesiology Department for an EBP. With the patient resting in a left supine position, an 18-gauge Tuohy needle was inserted into the middle epidural compartment at the lumbar level L_2_–L_3_. Blood was collected from the patient’s left basilic vein. A total of 35 mL of autologous blood was injected into the epidural space via an extension pipe under strict aseptic conditions. The patient was advised to stay supine for 24 h and reported immediate relief of symptoms afterwards with a simultaneous reduction of the dizziness. One month later at the follow-up, the patient remained asymptomatic and significant radiological improvement in the thickness of the subdural hematomas was also demonstrated compared to the previous examination; the left frontal one decreased to 5.5 cm × 3 mm and the right temporal–parietal one decreased to 3.5 cm × 4 mm. Their imaging was accompanied by elimination of pachymeningeal enhancement around the cerebral hemispheres on the flair sequence (Figure 1c).

### 2.2. Patient II 

**Figure 2:**
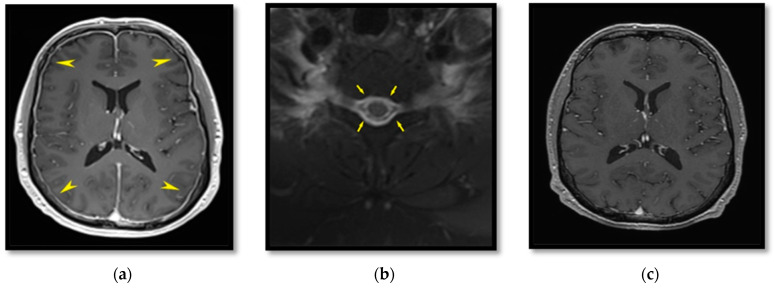
T_1_W sequence in transverse plane, after intravenous administration of contrast, which causes the vascular structures to appear whiter and any pachymeningeal enhancement to be depicted more distinctively. Meningeal enhancement is depicted (yellow arrow heads) (**a**). T_2_W sequence in transverse plane at the cervical level; the presence of epidural fluid is highlighted (arrows) (**b**). T_1_W sequence in transverse plane after intravenous contrast administration; there is a distinct reduction in the meningeal thickening and strengthening compared to the first imaging (**c**).

A 38-year-old-male came to the attention of the Neurology Department with complaints of an orthostatic headache of three-week duration and pain localized at the cervical segment of the spine and radiating to the frontal region accompanied with vomiting during the first days. He also complained about hearing impairment and diplopia at all sight positions. He did not report any history of trauma. His neurological examination revealed paresis of ocular muscles (especially at the right side), decreased sensation of the maxillary (V_2_) and the mandibular (V_3_) branches of the trigeminal nerve at the left side of the face and posterior neck pain and stiffness. Radiologic findings of magnetic resonance of the brain showed significant enhancement of the meninges of brain hemispheres and cerebellum (Figure 2a) and a small right parietal superficial venous thrombosis that remained stable without extending further; this was considered as a complication of SIH. Additionally, magnetic resonance of the spine did not reveal any sign of CSF leakage, but did reveal an extensive layer of epidural fluid collection along the spine from the cervical (Figure 2b) to the lumbar level. The diplopia was considered a result of paresis of the VI cranial (abducens) nerve.

The lumbar puncture test showed that the CSF pressure was 6 cm H_2_O. Taking into consideration the clinical and radiological findings of the patient, SIH was suspected; thus, the patient was referred to the Anesthesiology Department for an EBP. A total amount of 30 mL of autologous blood was injected to the epidural space at the lumbar level L_1_–L_2_ under strict aseptic conditions. The procedure was well tolerated and the patient reported gradual improvement of the headache and the diplopia from the first hours after the EBP. The following day, he was discharged from the hospital and placed on a regimen of anticoagulants as treatment for the superficial venous thrombosis; a thrombophilia test was ordered, while no physical activity and good hydration for three weeks were advised. An MRI performed one month later showed improvement of the described extradural fluid collection and of the enhancement of the thickened dura (Figure 2c). The patient was back to his previous physical activities with no remaining symptoms.

### 2.3. Patient III

**Figure 3:**
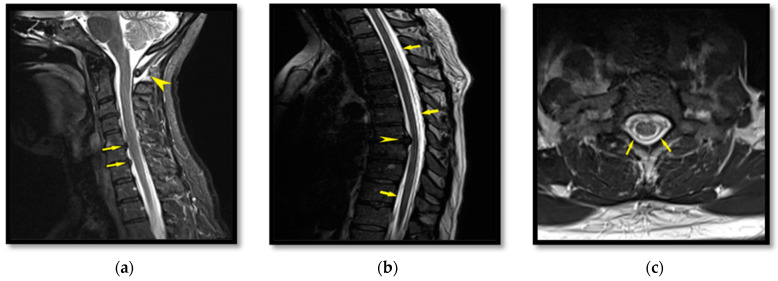
T_2_W sequence in sagittal plane at the cervical level; collection of fluid under the occipital level is highlighted by the yellow arrow head. Posterior projections of the C_5_–C_6_ and C_6_–C_7_ intervertebral discs with pressure on the meningeal sac are depicted by the small yellow arrows (**a**). T_2_W sequence in sagittal plane at the thoracic level demonstrating presence of extradural fluid collection (small yellow arrows) and posterior projection of T_7_-T_8_ intervertebral disc with compression of the meningeal sac (yellow arrow head) (**b**). T_2_W sequence in transverse plane at the thoracic level; the extradural fluid collection appears white and is highlighted by small yellow arrows (**c**).

A 44-year-old-female presented to the Neurology Department complaining of an intense frontal –occipital headache and working disability; her symptoms persisted in spite of conservative treatment. As with the other cases, the headache was present when she was standing and was relieved in the horizontal position. No other remarkable neurological symptoms were detected. Spine MRI imaging showed extradural fluid collection extending from the cervical level C_6_ (Figure 3a) to the lower thoracic level (Figure 3b,c). Fluid collection was attributed to dural tears from disc protrusions, which were obvious at both the cervical and the thoracic level. Lumbar puncture test revealed a CSF pressure of 5 cmH_2_O.

MRI findings in combination with the clinical features were consistent with the diagnosis of SIH. Consequently, the patient was also referred to the Anesthesiology Department for an EBP. Under aseptic conditions, 43 mL of autologous blood was withdrawn from her right basilic vein and was gradually injected at the thoracic level T_11_–T_12._ This amount of autologous blood was well tolerated and after 24 h of strict bed stay, the patient felt significant relief from her symptoms and reduction of the headache intensity. One month later at the follow-up, she had returned to all her physical activities and had resumed her work ability. As with the previous cases, the MRI findings at the follow-up revealed regression of the pathological extradural fluid collection.

In summary, in this case series, we report the efficacy of EBP as a therapeutic option in SIH. A summary of the patients’ characteristics is presented in Table 1. All patients were managed in the Anesthesiology Department and the Neurology Department of the Medical School of the National and Kapodistrian University of Athens, Greece. Written informed consent from the patients was obtained before publication of this report and any accompanying images.

## 3. Discussion

SIH is a rare syndrome with an annual incidence of approximately 5/100,000, peaking around the fourth or fifth decade of life and being slightly more common in women [8]. The clinical presentation consists of symptoms of a debilitating positional diffuse headache, occipital or frontal, typically worsening upon resumption of the upright posture, improving when recumbent and being relieved within 15–30 min of lying down or with other maneuvers that increase intra-abdominal pressure [9]. However, orthostatic headache may not be invariably present. Other associated symptoms include nausea, vomiting, posterior neck pain or stiffness, diplopia, blurred vision, photophobia, tinnitus, other subjective hearing disturbances or rarely even a comatose condition resembling encephalopathy [10]. The onset of headache is often sudden and patients can recall the specific time symptoms started. There are two presumed mechanisms behind the development of headache in SIH: first, the CSF leakage through the dural tear and brain sagging leads to stretching and stimulation of pain-sensitive sensory cranial nerve fibers due to the downward shift of the brain; secondly, the loss of CSF leads to cerebral vasodilatation as a compensatory adenosine-mediated mechanism to maintain intracranial volume according to the Monro–Kellie doctrine, which states that the sum of volumes of the brain, CSF and intracranial blood volume remains constant [11]. Therefore, any decrease in CSF volume should result in a compensatory increase in the volume of cerebral blood in light of unchanged intracranial volume [12]. This mechanism is further supported by the beneficial effect of vasoconstrictor medications, such as caffeine and theophylline. Our patients’ main complaint was not only the headache; one of them reported intense dizziness, which is an uncommon clinical presentation of the syndrome.

Although the true pathophysiology remains unclear, it is likely in most patients that a tear in the dura matter allows CSF leak and subsequent intradural CSF hypovolemia [13,14]. Symptoms can develop without any demonstrable cause (preceding trauma, history of central neuraxial block, iatrogenic causes) and theories about connective tissue disorders, malnutrition, short stature or female predominance due to hormones remain unproven. Although there was a theory that low CSF pressure was a main factor for the syndrome, nowadays it is supported that in most cases the pressure is normal, suggesting that insufficiency of CSF volume rather than CSF pressure is the underlying mechanism [15]. Recognized risk factors responsible for the pathophysiology of the syndrome are a weak meningeal wall or fragile meningeal diverticula that easily permit a CSF leak or dural tears often caused by calcified disk herniations and osteophytes at the thoracic and lower cervical level of the spine, which may have been the case in the third patient described [16]. Finally, another possibility might be the development of CSF–venous fistulas in cases where imaging shows no leakage, suggesting that in those cases CSF may be drained into the epidural veins [17]. In rare cases of patients with SIH symptoms and classical cranial imaging findings, with no evidence, however, of CSF leak in spite of repeated spinal neuroimaging, it has been hypothesized that a pathological increased compliance of the spinal compartment, perhaps due to a prolapsing arachnoid or increased distensibility of the dura mater, can cause orthostatic CSF shift from the cranial to the spinal region and the characteristic symptoms of intracranial hypotension and brain sagging [3]. Coexisting predisposing factors might be an overall low total CSF volume, smaller intracranial CSF volume, decreased CSF outflow resistance and decreased intracranial venous pressure, leading to increased CSF reabsorption [3]. 

Subdural hematomas are a serious complication of SIH that may lead to neurological deficits. They are attributed to traction of the fragile subdural bridging veins and compensatory vasodilatation due to low CSF pressure, causing the veins to bleed into the subdural space. Subdural hematomas can be managed by safely sealing off the dural tear or minimizing the CSF leak without evacuating them surgically, unless they lead to an acute change of consciousness [18]. Imaging in the first patient in our series showed the formation of two subdural hematomas, fortunately without any focal neurological deficits, which, however, might have occurred afterwards, had the case remained undiagnosed. Cerebral venous sinus thrombosis is another potentially fatal complication that can be due to stasis from cerebral vasodilatation which causes a hypercoagulable state in combination with damage of the cerebral venous endothelium as a result of a negative spinal–cranial pressure gradient [11]. The occurrence of any new neurological symptoms, including the development of non-postural headache, should create suspicion for the development of these serious complications. The second patient in our series had a small right parietal superficial venous thrombosis on imaging without any focal manifestations, which was attributed to SIH.

The diagnosis of SIH is based mainly on the orthostatic headache according to the third edition of the International Classification of Headache Disorders (ICHD-3) [19]. The patients in our series fulfilled the objective criteria of the diagnosis: an abnormal MRI of the brain and CSF leak at spinal imaging [20]. Additionally, their positive response to the EBP fulfilled the diagnostic criteria. Although CSF opening pressure was initially included in the basic criteria of the diagnosis, being an essential feature of SIH, nowadays it is controversial whether it is worth checking the opening pressure; it is expected to be low (<6 cm H_2_O), although most patients have CSF opening pressures within the normal range, making SIH a misnomer [21]. The first two patients in our series did not have CSF opening pressures <6 cm H_2_O; however, based on the clinical findings and MRI imaging (pachymeningeal enhancement and formation of subdural hematomas in the first patient as well as meningeal thickening and epidural fluid collection along the spine in the second patient), their diagnosis was undisputed. In fact, it is considered nowadays that SIH results from hypovolemia due to CSF leakage rather than CSF hypotension, and CSF pressure can lie within the normal range [17,22]. In other words, the demonstration of CSF pressure within normal range does not rule out the diagnosis of SIH, and since lumbar puncture can be challenging in the presence of shrunken dura (which is common in SIH) and might introduce further dural defects that could potentially exacerbate the leak, the diagnostic value of lumbar puncture becomes questionable and is not universally recommended for the verification of CSF opening pressure [15,23]. Furthermore, people with SIH may have normal CSF opening pressure first due to inadequate measurement and secondly due to the fact that lumbar puncture opening pressure is a snapshot method of measurement not reflecting the intracranial pressure in the upright position or CSF dynamics during postural changes [23]. Therefore, lumbar puncture should be undertaken with caution, taking into consideration its low sensitivity and the risk of worsening SIH. 

Imaging modalities which are readily available, non-invasive and easy to perform such as brain computerized tomography (CT) and brain MRI with contrast enhancement can reveal signs of intracranial hypotension, while the exact site of a CSF leak can be identified with spinal neuroimaging, such as spine MRI and MRI myelography. Invasive imaging studies, such as myelography, CT myelography and radioisotopic cisternography, belong to the past due to their risks, invasiveness and the advances in cross-sectional novel imaging techniques [24]. Classic cranial MRI changes described are diffuse pachymeningeal enhancement, engorgement of epidural venous plexuses, pituitary gland enlargement or hyperemia, extradural fluid extravasation, subdural collections and signs of brain sagging (drooping corpus callosum, convex pituitary superior margin, effacement of suprasellar cistern, reduced mamillopontine distance, reduced pontomesencephalic angle and crowding of tonsils at foramen magnum) [5,25]. However, these changes may not be observed in all cases and approximately 10% of patients with SIH may have normal brain imaging. Demonstration of the extradural presence of CSF on spinal imaging confirms the diagnosis and may also guide treatment attempts. Therefore, brain MRI helps in the initial diagnosis, whereas spinal MRI is useful in locating of the site of the leak. However, as mentioned before, occasionally no leak can be detected despite repeated diagnostic workup.

As mentioned before, the persistent leakage of CSF causes the characteristic headache of SIH, which may not respond to conservative treatment; this consists of complete bed rest with the head of the bed flat, fluid hydration, application of an abdominal binder, caffeine or analgesic medications. These conservative regimens aim at counteracting CSF loss and cerebral vasodilation. For example, fluid hydration is encouraged with the aim to increase CSF production; however, results are rather temporary and disappointing [26]. On the other hand, analgesic agents consist of non-steroidal anti-inflammatory drugs, opioids or medications commonly prescribed for tension headaches and migraine. Most of them will treat the compensatory vasodilation and not the presumed CSF leak. Caffeine and theophylline share two common mechanisms of action. First, they inhibit phosphodiesterase and antagonize adenosine, leading to cerebral vasoconstriction; second, they enhance CSF production by activating the sodium–potassium pump [26,27]. Cosyntropin and hydrocortisone are other proposed agents with proposed mechanisms of action in the expansion of blood volume (due to aldosterone-driven water retention), the increased production of CSF, the creation of edema in the dura which might seal the dural hole and finally the direct interaction with opioid receptors through an increase in brain β-endorphins [26,28]. Finally, migraine treating medications causing intracranial vasoconstriction have been tried, such as sumatriptan and methylergonovine, whereas gabapentin and pregabalin might also be partially helpful by inhibiting the sympathetic pathway of pain attributed to low CSF pressure [26]. The aforementioned medications may decrease pain severity scores; however, there is no compelling evidence for their efficacy. In 15–30% of cases, there may be spontaneous resolution of the symptoms or SIH may respond to only conservative measures within 1–2 weeks from the symptom onset. If the symptoms do not respond to conservative management, an EBP could be considered. The role of the EBP in this condition is well recognized and relieves the symptoms in up to 80–90% of patients, being reported to be successful the first time in the majority of cases [23,24]. An EBP is the injection of autologous blood into the epidural space, aiming at sealing off the dural tear and discontinuing CSF leak. Two possible mechanisms can explain the effectiveness of this treatment: the tamponade effect from the injected blood volume causing compression of the thecal space and a quick increase of lumbar and consequently intracranial CSF pressure, as the CSF is displaced to the more cephalad cranial compartment (immediate effect), and plugging of the dura opening with the creation of a blood clot brought about by interaction between the injected blood and procoagulant components in leaking CSF (long-lasting effect). In fact, the aforementioned increase in intracranial pressure after the performance of an EBP has been shown to be short-lived: compression of the thecal sac is not sustained and does not explain its prolonged effect, since radiological studies have shown that the epidural blood remains in situ for up to 18–24 h [29]. Additionally, animal studies have shown that seven days after the performance of an EBP, there is diffuse fibroblastic activity and collagen formation [30,31]. Therefore, the definitive repair is most probably facilitated by the inflammatory reaction of the dura and scarring in the epidural space, which is activated by the injected blood. The EBP can be performed either at the exact site of the CSF leak (also referred to as “targeted-EBP”) or at the lumbar level (also referred to as “blind EBP” or “non-targeted EBP”), where there is less risk for damaging the spinal cord in case of accidental dural puncture. Targeted EBPs at the exact site of the leak are considered more successful than the blind ones, though the injected blood can reach the site of the leak as far proximally as the cervical region, so the chance of success is substantial even after a blind EBP [32]. A recent meta-analysis demonstrated similar proportions of success rates for both the targeted and non-targeted techniques [23]. The literature supports that most of the spontaneous CSF leaks occur at the cervico-thoracic level or all along the thoracic segment of the spine. All of our patients had disabling postural headache or dizziness hindering their daily activities and compromising their quality of life. They were all subjected to an upper lumbar or a lower thoracic EBP regardless of the exact site of leak, since research has shown that the blood injected during an EBP preferentially spreads in the cephalad direction, as already mentioned. They all reported immediate or gradual resolution of their symptoms, while significant imaging improvement was displayed on diagnostic follow-up. This is in accordance with the literature reporting identical rates of success and lack of complications [15,32,33,34].

As for the procedure itself, written informed consent should be obtained from the patient, as well as documentation about the discussion on the risks, benefits and availability of alternative treatment. Strict aseptic conditions should be adhered to, including for the procedure of blood withdrawal. The procedure should not be performed in case of systemic or in situ infection or if there is overt coagulopathy. There is no consensus on the optimal volume of blood required. It has been shown that a larger amount of injected blood predicts a better response to the first epidural blood patch [23,35]. Therefore, most clinicians agree that 20–30 mL of blood is more likely to guarantee success while others aim for 30 mL and discontinue administration as soon as the patient complains of back pain. The patients in our series tolerated the administration of 30–43 mL of blood injected. Most practitioners perform the procedure with the patient in the lateral decubitus position, as we did in our series, while few attempt the sitting position. An alternative approach is to perform the EBP and tilt the patient in a 30° Trendelenburg position during and for the next 16 h after the procedure in order to allow the blood clot to migrate to upper segments of the spine and seal up the leaks [33]. It is also advocated by some centers to place the patient in the Trendelenburg position for at least one hour before the blood injection. The rationale is, apart from pain relief, to reduce the flow of CSF in the spinal region, minimizing the volume of CSF in the spinal epidural space that may dilute injected blood and favoring a collapse of the dura mater and the approach of the edges of the dural hole, thus facilitating the sealing of the hole [33,34]. Intense exercise and excess movements post-procedurally should be discouraged to reduce the risk of dislodgement of the blood clot. At the conclusion of the procedure, our patients were asked to lie still for approximately two hours to facilitate clot formation at the site of the dural tear and were then allowed to walk.

As already mentioned, all our patients reported complete resolution of their symptoms both during the first hours after the EBP and also one month later at the follow-up without any untoward events. Factors associated with better outcome after the performance of the EBP include older age, absence of tinnitus, intense pachymeningeal enhancement and subdural collections and a larger volume of blood (>20 mL) used. Apparently, sex and body mass index bear no correlation to the success of the EBP [6]. An EBP is generally a safe procedure but may be occasionally associated with complications. Back pain is commonly reported afterwards but is rather mild and self-limiting. Meningitis and septic complications have been reported and are most probably attributed to poor aseptic technique during the procedure. Delayed radicular symptoms have been described, most probably attributed to irritation of nerve roots by by-products of the injected blood, while another occurrence might be rebound intracranial hypertension, which usually resolves within a few days. There are also very rare reports of facial nerve palsies, chronic adhesive arachnoiditis, subdural or spinal hematomas, permanent spastic paraparesis or cauda equina syndrome; therefore, thorough follow up and clear communication about alarming symptoms is deemed necessary [5,23].

Apart from EBP, which is considered the gold-standard of therapy, other alternative pain interventions have been attempted, though with limited evidence as yet. Greater occipital nerve block is one of these and seems to act by interrupting the pain transmission to the trigeminus nucleus caudalis (which is activated by dural stretch) and by reducing central sensitization [36]. Another intervention is the sphenopalatine ganglion block, which is believed to act by blocking sympathetic outflow to the cerebral vasculature, thus diminishing cerebral vasodilation [37]. There also have been reports of the use of other epidural solutions as alternatives to EBPs, such as epidural saline, epidural dextran, epidural hydroxyethyl starch or epidural gelatin, though without sound evidence to support their use, since the increased pressure in the epidural space that they cause will not be sustained [22]. There are also reports of cases of SIH successfully treated by epidural patching with fibrin glue [38]. Finally, there is the option of surgical closure of the dural perforation in cases of CSF leaks which have been clearly identified by spinal imaging. Surgical interventions include epidural packing, primary dural repair or clipping of the leaking root sleeve [5,6,7,39]. However, if the site of the CSF leak cannot be properly localized, surgical treatment options are unfortunately limited.

To sum up, the target of the therapy in cases of SIH is either to increase the CSF pressure or to seal the CSF leak. Taking into consideration the inefficacy of conservative treatment, the debilitating symptoms of SIH and the potential for life-threatening complications, early EBPs are warranted. The literature suggests that the success rate of the first attempt of an EBP ranges between 80 and 90%. In case of persistent symptoms, one might consider repeating the EBP up to two or three times with an interval time of 5 days between the repeated EBPs to limit the risk of spinal cord compression due to excessive EBP volume. However, in case of patients refractory to treatment with EBP headache, the physician should be alerted to review the differential diagnosis [14].

## 4. Conclusions

In conclusion, this report is a reminder that SIH may be an under-diagnosed or misdiagnosed syndrome with a variety of imaging features as well as a great diversity of signs and symptoms at presentation not limited to the classic orthostatic headache. Therefore, the diagnosis should not be excluded based on the presence of non-orthostatic headache, normal neuroimaging studies or normal CSF opening pressure. The procedure of an EBP secures the diagnosis and remains the main treatment modality in cases of severe SIH syndrome refractory to conservative management. The majority of reports agree that the EBP should be attempted early in patients failing conservative treatment even if the exact site of the leak cannot be localized. The current case series describes not only the persistent symptoms of the patients but also reports the formation of a SDH, which is a severe complication of SIH. Although the exact etiology of SIH is not known, it is believed to be due to a CSF leak which produces the clinical spectrum. Brain MRI and spine MRI with contrast should be offered as first line investigations in patients with clinical suspicion of SIH and help in demonstrating indirect signs of a low volume of CSF, though it may not be possible to locate the actual site of the leak. In our series, EBPs were performed in order to restore CSF volume; they relieved the patients’ symptoms, suggesting that EBP is a well-accepted and beneficial treatment modality for SIH when conventional measures fail.

## Figures and Tables

**Table 1:** Characteristics of the patients involved in the current case series.

	Patient I	Patient II	Patient III
Sex	Male	Male	Female
Age (years old)	53	38	44
Main symptoms	Dizziness	Headache, diplopia	Headache
Subdural hematoma	Yes	No	No
EBP level	L_2_–L_3_	L_1_–L_2_	T_11_–T_12_
Amount of autologous blood infused (mL)	35	30	43
Complications	None	None	None
Response to EBP	Yes	Yes	Yes

## Data Availability

The datasets generated during and/or analyzed during the current study are available from the corresponding author on reasonable request.
